# The Regulation of Lymph Node Pre-Metastatic Niche Formation in Head and Neck Squamous Cell Carcinoma

**DOI:** 10.3389/fonc.2022.852611

**Published:** 2022-04-27

**Authors:** Chen Hu, Qiang Huang, Qing Sun

**Affiliations:** ^1^ Department of Otorhinolaryngology, QingPu Branch of Zhongshan Hospital Affiliated to Fudan University, Shanghai, China; ^2^ Department of Otorhinolaryngology, Eye & ENT Hospital, Fudan University, Shanghai, China

**Keywords:** lymphangiogenesis, extracellular vesicles, pre-metastatic niche, head and neck squamous cell carcinoma, prognosis

## Abstract

In many distinct forms of malignancies, there is a close relationship between lymph node (LN) metastases and further dissemination to distant organs, and this is a critical prognostic factor. At the beginning of the process, the original tumor secretes soluble substances or releases extracellular vesicles (EVs) that are carried through lymphatic channels to draining (sentinel) LN. The tumor-derived factors then drive LN remodeling. These significant alterations occur prior to the emergence of the first metastatic cell, bringing about the development of a pre-metastatic niche that allows metastatic cells to survive and thrive. In this review, we discuss current information available about the regulation of lymph node pre-metastatic niche in head and neck squamous cell carcinoma (HNSCC), and the role of EVs in forming the pre-metastatic niche.

## Introduction

Head and neck squamous cell carcinoma (HNSCC) is the most common head and neck cancer, and arises from the mucosal epithelium of the oral cavity, pharynx, and larynx (1). Patients with HNSCC are at risk of cervical lymph node (LN) metastases. Cervical LN involvement is a well-known prognostic marker for HNSCC, and the presence of LN metastases is thought to be a predictor of poor patient outcomes (2, 3). Specifically, high levels of lymphangiogenic growth factors and high lymphatic vessel (LV) density in individuals with cancer indicate that LN metastases are more likely to occur, and the prognosis is usually poor (4, 5).

To facilitate the development of new LVs (lymphangiogenesis), tumor cells produce growth hormones, RNA, and cytoplasmic proteins, and growth factors. These tumor-derived factors can create microenvironment around the organs in which metastases might consequently occur. This advance preparation of the target organ microenvironment is thought to facilitate the tumor cells’ survival and multiplication at the distant site.

This review outlines recent achievements of the regulation of lymph node pre-metastatic niche formation in HNSCC, and the role of EVs in forming the pre-metastatic niche was also discussed.

## The Lymph Node Pre-Metastatic Niche Formation in HNSCC

According to the early studies of Isaiah Josh Fidler et al. (6, 7), metastasis was determined by the structure of the arterial and lymphatic pathways that drain the primary tumor, although tumor cells reached the vasculature of all organs, metastases selectively formed in certain organs but not others. Later related researches (8, 9) have showed the interplay among the microenvironment of the primary and metastatic organs: in order for tumor cells to engraft (metastatic niche) and flourish in secondary locations, a suitable microenvironment (pre­metastatic niche) must form. These niches are formed by tumor-secreted substances and can be either freshly generated or modifications of pre­existing physiological niches. Psaila and Lyden have proposed the notion of a pre-metastatic niche (10), their groundbreaking research showed that tumor cells shed or secrete substances that create a circumstance-related metastasis. These growth factors and chemokines/cytokines produce a distinct milieu that promotes metastatic progression: pre-metastatic niche formation, and LN metastasis (4, 11–13).

Lymph node metastases (LNM) are common over the course of many cancers, and their presence often indicates a bad prognosis. The lymphatic system’s pre-metastatic conditioning of the microenvironment in lymph nodes (so-called lymph node pre-metastatic niche), which makes them receptive and supportive metastatic habitats for disseminated tumor cells, is aided by the discharge of tumor-derived substances such as antigens, growth factors, cytokines, and exosomes (12). HNSCCs, like many other cancers, spread through the lymphatic system (14–18). Indeed, sentinel LNs are the first draining LNs where metastases occur, and they are thought to be a predictor of poor patient outcomes (19). In several types of solid tumors, the level of lymphangiogenesis and the density of LVs are related to LN metastasis and the prognosis of patients (20, 21). For HNSCC, this link has been verified numerous times (22–24). These results show that lymphangiogenesis occurs before tumor cells arrive in the metastasis locations of patients with HNSCC. Tumor-derived signals travel from the lymphatics to the draining LN, where they stimulate the formation of localized LVs. As a result, the enlarged lymphatic network in tumor-free lymph nodes is a very early pre-metastatic indicator. Family members of vascular endothelial growth factors (VEGF) and other non-VEGF-mediated molecular are able to induce lymphangiogenesis in tumor (25), and their specific mechanisms in regulating lymphangiogenesis in HNSCC are discussed in the following sections (**Table 1**).

**Table 1:** The role of different molecular in inducing lymphangiogenesis in head and neck squamous cell carcinoma (HNSCC).

Growth factors and chemokines/cytokines	Member/receptor	Specific mechanism in lymphangiogenesis	Reference
Vascular endothelial growth factors (VEGFs)	VEGF-A	Targeting with VEGFR-2 and enhancing lymphangiogenesis	(26)
	VEGF-C	Targeting with VEGFR-3 and enhancing lymphangiogenesis	(27)
	VEGF-D	Targeting with VEGFR-3 and enhancing lymphangiogenesis	(28)
Periostin	Integrin-αvβ3	Periostin itself as well as periostin-induced upregulation of VEGF-C promote lymphangiogenesis	(27)
Angiopoietins	Ang-1	Inducing high levels of VEGFR-3 and enhances effectiveness of VEGF-C and VEGF-D in lymphangiogenesis	(29)
	Ang-2	Specific association with lymphangiogenesis in HNSCC is not clear so far	(30)
Laminin γ2	Integrin-α3	Regulates integrin-α3-dependent EVs uptake	(31)
Insulin-like growth factor (IGF)-1/2	IGF-1R	Inducing lymphangiogenesis stimulating phosphorylation of extracellular signal-regulated kinase (ERK), Akt, and Src	(28)

### Lymphangiogenesis Mediated by Vascular Endothelial Growth Factors (VEGFs)

Secreted growth factors have been shown to be major regulators of lymphangiogenesis, regardless of their source. Angiogenesis is the process by which new blood vessels develop from primary vessels, and vascular endothelial growth factors (VEGFs) are considered essential elements in angiogenesis (32). The role of VEGF family proteins in angiogenesis and tumor formation is well understood (33, 34).

**Figure 1:**
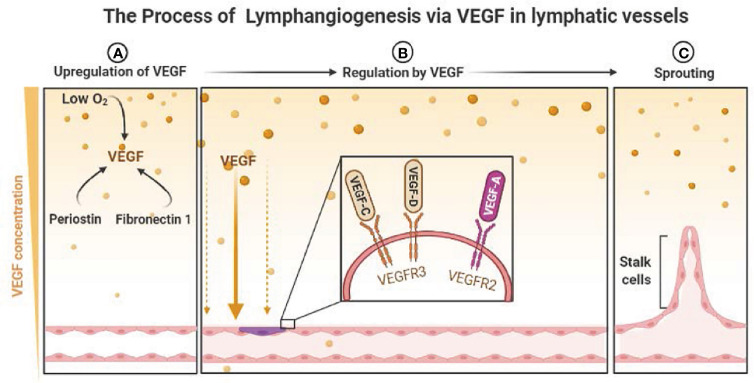
The Process of Lymphangiogenesis *via* VEGF in lymphatic vessels. **(A)** Low O_2_ and some molecular induce the upregulation of VEGF. **(B)** The regulation of lymphangiogenesis by the link of VEGFs and VEGFRs. **(C)** The sprouting of lymphatic vessels. (P.S. Created with https://BioRender.com).

VEGF-C and VEGF-D have angiogenic capabilities in lymphangiogenesis, and they are the most widely researched components to date (35). These two molecules promote lymphangiogenesis by linking with the receptor VEGFR-3, which is mostly expressed in lymphatic endothelium cells (LECs) and monocytic hematopoietic cells in adults (36). When VEGFR-3 is stimulated in LECs, a cascade of signals causes the cells to expand and migrate, protecting them against apoptosis (37–39). Furthermore, VEGF-A can cause LV growth by activating the receptor VEGFR-2: it was found that original tumors with high levels of VEGF-A induced lymphangiogenesis in sentinel lymph node (SLN) before LN metastases (40). Higher levels of these three VEGF have been linked to the density of LVs and LN metastases and poor prognoses in patients with HNSCC (41, 42), and there are some molecular can induce lymphangiogenesis in HNSCC by regulating the expression of VEGF (**Figure 1**).

In a study by Kudo et al. (27), periostin enhanced lymphangiogenesis by increasing the tumor secretion of VEGF-C and diffusion and tube creation in LECs *via* Src and Akt activation. Importantly, periostin itself can directly promote lymphangiogenesis by binding integrin-αvβ3. And according to their result, serum periostin levels in patients with HNSCC were found to be strongly linked to VEGF-C levels and malignant characteristics, such as advanced tumors and LN metastases. Ching-Chia Lin et al. (43) have demonstrated that WNT1-inducible signaling pathway protein-1(WISP-1) can promotes VEGF-C expression in OSCC cells through the ILK/Akt pathway and WISP-1 induces VEGF-C production by inhibiting miR-300 expression, which lead to VEGF-C-dependent lymphangiogenesis. Moreover, research of Yoshihiro Morita (44) demonstrates that elevated expression of cellular Fibronectin 1 (FN1) and following activation of focal adhesion kinase (FAK) lead to increased VEGF-C expression, lymphangiogenesis, LNM and promoted epithelial-mesenchymal transition (EMT) in OSCC cells and suggest that FN1-phosphorylated FAK signaling cascade is a potential therapeutic target in the treatment of LNM in OSCC (44).

Furthermore, research of Hu An et al. (45) showed that sirtuin 2 (SIRT2) inhibits hypoxia-induced VEGF-D synthesis in head and neck cancer (HNC) cells, and expression of SIRT2 was significantly linked to VEGF-D expression and lymphangiogenesis in HNC tissue, where a substantial fraction of SIRT2 protein was produced at a lower level. They have investigated SIRT2 mediated regulation of VEGF-D expression and lymphangiogenesis by deacetylating endothelial PAS domain protein 1 (EPAS1). However, in individuals with HNC, increased SIRT2 levels are associated with a worse prognosis.

In addition, VEGF-A has been shown to induce lymphangiogenesis and SLN metastasis of OSCC cells *in vitro*, and 3-O-acetyloleanolic acid (3AOA) has been shown to inhibit tumor growth, tumor-induced lymphangiogenesis, and SLN metastasis in a VEGF-A-induced oral cancer sentinel lymph node (OCSLN) animal model created with high expression of VEGF-A in squamous cell carcinoma (SCCVII) cells (26). The anti-lymphangiogenic effects of 3AOA are mediated *via* suppression of VEGF-A/VEGFR-1 and VEGFR-2 signaling.

Despite the fact that lymphatic spread is an important event in the progression of HNSCC, the levels of VEGF-C, VEGF-D, and VEGFR-3 are not related to the clinicopathological characteristics reported in other studies, suggesting that lymphangiogenesis in HNSCC is mediated by other signaling pathways (46).

### Lymphangiogenesis Mediated by Non-VEGF Means

To identify non-VEGF-mediated molecular mechanisms that lead to tumor lymphangiogenesis and LN metastasis, a plethora of other growth factors have been researched (28) (**Table 1**).

In one study, VEGFR-3 expression increased in LECs after treatment with angiopoietin-1 (Ang-1), implying that Ang-1 promotes lymphangiogenesis by making lymphatic capillaries more sensitive to VEGF-C or VEGF-D (29). In another study, overexpression of Ang-1 and Ang-2 was linked to a worse prognosis in OSCC (30). However, studies on the potential role of Ang-2 in the lymphangiogenesis of HNSCC are lacking.

Wang et al. discovered that the secreted EVs in patients with OSCC with LN metastases was considerably elevated (31). Low expression of laminin-332 in LN1-1 cells reduce EV-mediated LEC migration, lymphangiogenesis, and LN metastasis. The study showed that knocking down integrin-3 resulted in a decrease in the role of laminin-γ2-enriched EVs, implying that integrin is required for EV uptake by LECs.

Furthermore, insulin-like growth factor (IGF)-1 and IGF-2 can promote a signaling pathway that is distinct from that induced by the VEGF-C/VEGF-D–VEGFR-3 system, showing that these two growth factors have direct lymphangiogenic activity (28). Both substances promote LEC proliferation and migration by phosphorylating intracellular signaling components (47), which can lead to lymphatic dissemination, metastasis, and tumor recurrence. Enhanced IGF-1 receptor (IGF-1R) expression has been observed in initial undifferentiated oropharyngeal and nasopharyngeal cancers, as well as in LN metastases (48), and is related to high metastasis and recurrence rates (49).

Additionally, Vyomesh Patel et al. (50) show that the activation of mTOR is a critical event which induces lymphangiogenesis in HNSCC. Furthermore, the prolonged treatment with rapamycin and rapalogRAD001 diminished the dissemination of HNSCC cancer cells to the cervical lymph nodes in a newly developed orthotopic HNSCC model, thereby prolonging animal survival. Thus, the use of mTOR inhibitors may represent a novel molecular-targeted approach for metastasis prevention in patients with HNSCC. In the study of SATOMI ARIMOTO et al. (51), their results clearly showed that podoplanin and lymphatic vessel endothelial hyaluronan receptor 1(LYVE-1) were expressed in most of the OSCC cases and were strongly associated with lymphangiogenesis. Podoplanin and LYVE-1 may be used for predicting lymphatic status in OSCC in the future, while there is no consensus, yet. Moreover, Jiajia Li et al. (52) have discovered that 6-phosphofructo-2-kinase/fructose-2,6-biphosphatase 3 (PFKFB3) was correlated with lymphangiogenesis in OSCC, PFKFB3 may promote LNM by regulating the expression of podoplanin (PDPN). However, the real role of PFKFB3 in lymphangiogenesis remains need further researches (52).

## The Regulation of Pre-Metastatic Niche Formation by Extracellular Vesicles (EVs)

### The Structure, Content, and Properties of EVs

EVs are lipid bilayer particles with dimensions ranging from 30 nanometers to several micrometers (53), and they comprise a heterogenous population, with differences in biogenesis, size and contents between different subpopulations. Based on these parameters, EVs are generally classified as large (large extracellular vesicles (lEVs)), including apoptotic bodies, large oncosomes and microvesicles; small (small extracellular vesicles (sEVs)), including exosomes; or extracellular particles (EPs), including exomeres and chromatimeres (54). The term “extracellular vesicle” or “EV” has now been agreed on by the international community as the consensus generic term for lipid bilayer-delimited particles released from the cell (55, 56). EVs contain proteins, messenger RNA (mRNA), and noncoding microRNA (miRNA) (57, 58), the composition of an EV depends on the cell type and the physiological/pathological setting, the pathological setting can also influence the cytokine profile associated with EVs (59, 60). EVs were once assumed to be a route for cells to dispose of unwanted materials; however, recent research has shown that EVs are crucial for strictly regulated bidirectional communication (61, 62). EVs have recently attracted considerable attention because their molecular/genetic profiles have been found to be similar to those of the original cells (63, 64). Moreover, due to the selective sorting of cargo into EVs, the inherent features of EVs can differ from those of their cells of origin (65). EVs are secreted by nearly every cell, and their form, source, and molecular composition vary (66). Hence, EVs have potential as circulating biomarkers that carry information about the tissue-bound parent cells’ molecular composition and activity (67). For instance, if a cancer cell produces EVs, it may be possible to detect these cancer-derived EVs in the plasma and use them as an inspection index in the diagnosis of cancer (68, 69).

### The Role of Tumor-Secreted EVs in the Pre-Metastatic Niche Formation

Organotropism is a condition that describes how circulating tumor cells homing to certain organs as a result of complicated tumor–stroma interactions, however, the exact potential mechanism is not clear yet (70). According to other researches, sEVs may play a critical role in organotropism. sEVs not only recruit bone marrow-derived cells, endothelial progenitor cells, and mesenchymal cells to generate an appropriate niche environment, but they also cause the overexpression of proinflammatory chemicals and facilitate vascular leakiness (10, 71). Prior to the entrance of cancer cells, these changes in distant organs have already happened. Interestingly, melanoma sEVs tend to move to sentinel lymph nodes, breast cancer cells sEVs to the lung, and pancreatic cancer cells sEVs to the liver, according to multiple studies (72–74). Thus, the questions arise of why and how sEVs are directed to specific sites to enable organotropic metastasis may explain the role of EVs in the lymph node pre-metastatic niche formation.

It has been demonstrated that tumor-secreted EVs can communicate with neighboring non-tumor cells (75, 76), even they can transport oncogenic molecules to normal cells which lead to development of tumor (60). Ferdinando Pucci et al. (77) have showed that endogenous tumor-secreted EVs efficiently disseminate *via* lymphatics in mice and humans. And tumor-secreted EVs induce vascular leakiness and facilitate circulating tumor cell arrival to distant sites, accumulating researches confirm that vascular leakiness is considered a hallmark of pre-metastatic niche formation (10, 60, 78). EVs have been extensively explored and well documented in the literature in terms of how they regulate pre-metastatic niche development in various cancers (73). For instance, it has been found that sEVs derived from metastatic melanoma cell lines are rich in nerve growth factor receptor, and can enhance lymphangiogenesis, tumor cell adhesion, pre-metastatic niche formation, and LN metastasis (79); Noelle Leary et al. identify EVs-mediated melanoma—LN LEC communication as a new pathway involved in tumor progression and tumor immune inhibition (80). And the study of Li et al. showed that exosomal CXC chemokine recepter-4 from Hca-F cells promoted LECs proliferative rate and lymphatic tube formation ability (81). The lymphatic network remodeling may guide tumor metastasis in SLNs, and sun et al. found that CT26 cell exosomes promote the proliferation of lymphatic endothelial cells and the formation of lymphatic network in SLN, facilitating the SLN metastasis of colorectal cancer, which demonstrates tumor-derived exosomes can modify the microenvironment in adjacent organs and initiate a premetastatic niche (82). Moreover, Zhou et al. identify that cervical squamous cell carcinoma-secreted exosomal miR-221-3p promotes lymphangiogenesis and lymphatic metastasis by targeting vasohibin-1-secreted exosomal miR-221-3p transfers into lymphatic endothelial cells to promote lymphangiogenesis and lymphatic metastasis *via* downregulation of VASH1 (83).

Nowadays, HNSCC-derived EVs have received more and more attention (84). Cancer cells release EVs containing immunoregulatory factors, affecting the tumor microenvironment by mediating immune escape and playing a crucial role in the formation of the premetastatic niche (85, 86). And HNSCC is one of the most immunosuppressive human tumors, LNM is the most important prognostic determinant of HNSCC tumors in the survival rate of patients (87), and tumor-derived EVs and communication with the tumor microenvironment are critical factors in tumor metastasis (88). Apart from lymphangiogenesis, tumor-derived EVs can mediate the formation of pre-metastatic niche by other mechanisms in HNSCC. Chan et al. reported that EVs derived from nasopharyngeal carcinoma cells could markedly enhance the tubulogenesis, migration and invasion of human umbilical vein endothelial cells (89). Recent studies have shown that EVs rich in PFKFB3, MMP-13, intercellular cell adhesion molecule-1 or thrombospondin-1 can enhance the release of VEGF-A, IL-8 and then downregulate junction-related proteins, which promote tumor angiogenesis and vascular permeability and become a potential channel system for distant metastasis of tumor cells (89–91). The incidence of HPV (+) HNSCC has risen sharply in recent decades (92), while HPV (+) HNSCC responded better to treatment and had a significantly better prognosis than HPV (-) HNSCC (93). HPV (+) HNSCC EVs stimulated dendritic cells maturation and HPV (-) HNSCC suppressed it instead, which is critical for the good prognosis of HPV (+) HNSCC (94–96). Furthermore, it was found that the most abundant miRNA in HPV (+) EVs was miRNA-363-3p (97). Notably, in OSCC cells expressing miRNA-363-5p, cell proliferation decreased by 40–50% (98). These results suggest that intercellular communication mediated by HPV (+) EVs might play a dominant role in antitumor immune responses and inhibit tumor proliferation, which may provide a new treatment for HPV (+) HNSCC. According to the study of T. Whiteside et al., levels of PD-L1 carried by exosomes correlated with the lymph node status, and blocking of PD-L1+ exosome signaling to PD-1+ T cells attenuated immune suppression (99). Body fluids of patients with HNSCC are enriched in exosomes that reflect properties of the tumor, recent research of T. Whiteside et al. found that the purine metabolite levels in exosomes decreased in patients with advanced cancer and nodal involvement, their report provides the first evidence that HNSCC cells shuttle purine metabolites in exosomes, with immunosuppressive adenosine being the most prominent purine (100). Furthermore, related articles highlight the role of tumor-derived EVs in HNSCC: EVs mediate immune suppression and tumor progression by reducing the proliferation of CD8+ T cells and promoting the expansion, suppressive activity, and resistance of apoptosis of regulatory T cells (101).

**Figure 2:**
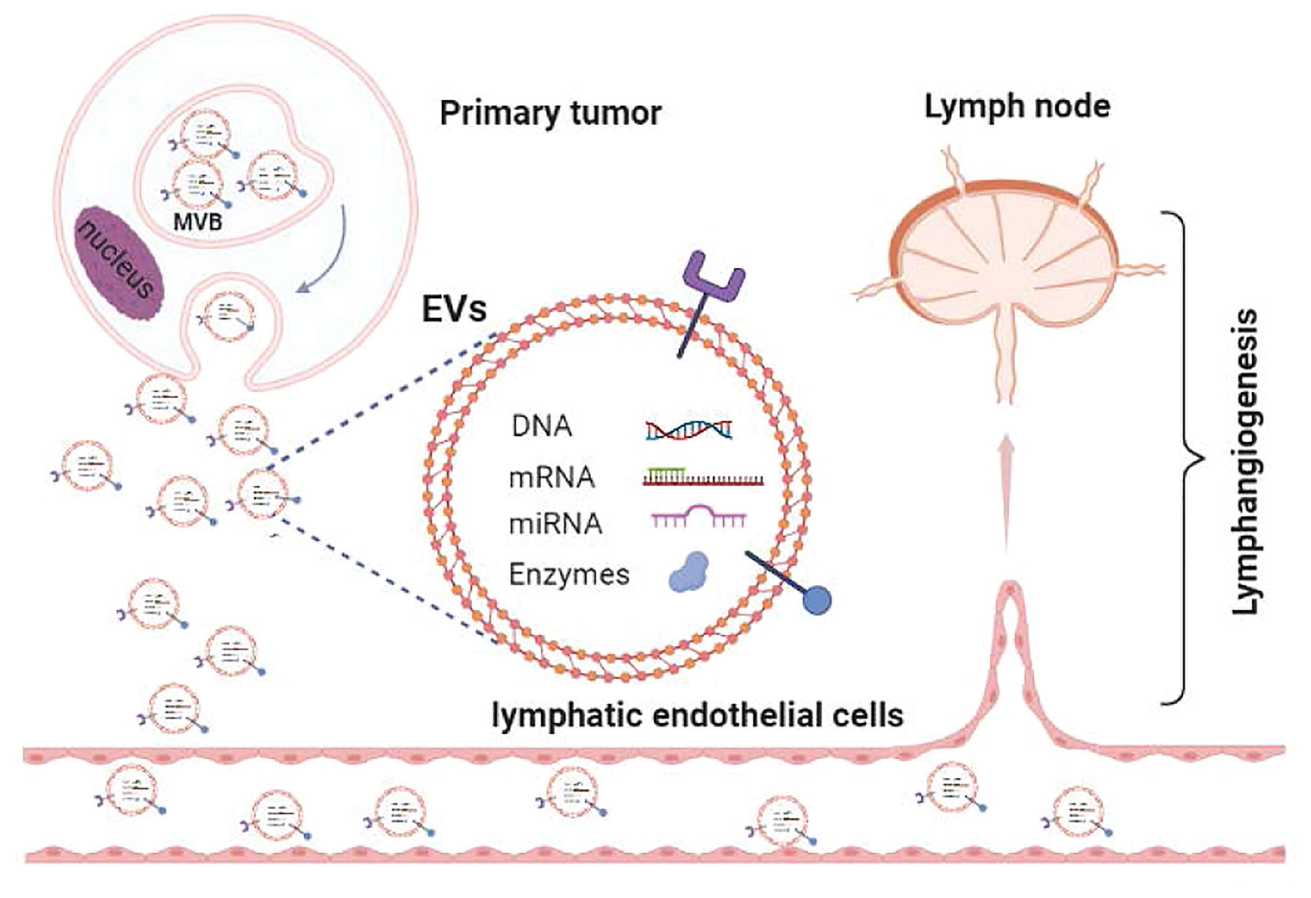
The role of EVs in regulating lymph node pre-metastatic niche formation in HNSCC. (P.S. Created with BioRender.com).

In summary, EVs work in a coordinated and planned manner to enhance tumor survival, re-educate immune cells, and generate pre-metastatic microenvironment (60). As a natural nanoscale vesicle, EVs can pass through the interstitial matrix entering the lymphatic circulation (102), which makes EVs ideal carriers for message transport between the lymphatic system and tumor cells, then prepare a lymph node pre-metastatic niche for HNSCC metastasis (**Figure 2**). Future research is needed to better understand the features and mechanisms driving EV production, trafficking, and uptake that are particular to HNSCC to fully comprehend their impact on disease development and progression. To completely correlate cause and function, an understanding of the presence of EV subpopulations is also required. For example, a recent study identified a new type of EV, the supermere, that has potential as a circulating biomarker and therapeutic target for a variety of diseases in the future (103).

## Therapy Based on Anti-Lymphangiogenesis Strategy

Given that lymphangiogenesis plays a critical role in LN metastasis, it may be viable to treat cancers using an anti-lymphangiogenesis strategy (104). Some receptor tyrosine kinase inhibitors may exert negative effects on tumor-related lymphangiogenesis (and LN and distant organ metastases) by targeting the VEGF-C–VEGFR3 signaling pathway (105, 106). Many tyrosine kinase receptors are targeted by receptor tyrosine kinase inhibitors, yet it is difficult to determine which elements and pathways are involved in lymphangiogenesis.

A related study demonstrated that adipose stem cells treated with VEGF-C secreted exosomes, and this more efficient lymphangiogenic response revealed a potential therapeutic modality for increasing the efficiency of anti-lymphangiogenesis (107). Furthermore, it has been found that LECs with laminin-γ2-enriched EVs can improve lymphangiogenesis *in vitro*, and that the lymphangiogenesis resulting from the activity of EV-mediated LECs can be reduced by lowering the laminin-γ2 level (31). This unique approach to anti-lymphangiogenesis therapy *via* EV targeting requires additional research and validation. Furthermore, the angiostatic N-terminal 16 kDa fragment of human prolactin has been shown to induce apoptosis and inhibit lymphangiogenesis in microvascular dermal LECs (108). After treatment with the angiostatic fragment of prolactin, the density of the LVs of the primary tumor and the LNs was considerably reduced in a melanoma model. That study was the first to demonstrate that prolactin plays a critical role in lymphangiogenesis, and this finding could have implications for general therapeutic strategies (108). Related research demonstrated that a selective histone deacetylase (HDAC) 1/2 inhibitor (B390) not only restrains tumor growth by inducing apoptosis of tumor cells but also inhibits lymphangiogenesis and LV invasion *in vivo (* 109). Liu et al. found that overexpression of miR-320b is closely linked to peritumoral lymphangiogenesis and LN metastasis, and it is also worth mentioning that the miR-320b–PDCD4 axis activates the Akt pathway independent of VEGF-C (110). The anti-lymphangiogenesis strategy applying in the clinical treatment of HNSCC, however, not common so far.

On a more positive note, because EVs can traverse membranes in the tumor microenvironment by fusion and/or endocytosis (111), they have the potential to be used as biological drug delivery vehicles (112). For instance, tumor-derived EVs mediate the delivery of miRNA-9 to inhibit angiogenesis by targeting midkine gene and regulating the PDK/AKT pathway nasopharyngeal carcinoma. Additionally, the miRNA-9 levels in EVs were positively associated with overall survival, while midkine gene overexpression was positively correlated with poor prognosis in nasopharyngeal carcinoma patients. Thus, we can conclude that miRNA-9 can inhibit tumor angiogenesis, providing a new direction for anticancer treatment (113). In the future, EVs may play a significant role in cancer treatment of HNSCC.

The abovementioned studies show limitations of the anti-lymphangiogenic therapy in HNSCC based on the lack of studies that evidence its potential efficacy in this tumor type. While with further researches, anti-lymphangiogenesis may become a treatment option for HNSCC in the future.

## Conclusion and Perspective

The pre-metastatic niche is gradually being recognized as a tumor-induced circumstance that facilitates tumor cell dissemination and metastasis production. The development of a pre-metastatic niche is a complicated process, yet it is an essential step in the metastatic cascade, occurring prior to tumor cell colonization. Recent research conducted with numerous tumor models has identified many critical elements that play important roles in pre-metastatic niche formation. The present study has focused on LN pre-metastatic niche creation in HNSCC, which is dependent on lymphangiogenesis. However, paying attention to a single biological mechanism, such as lymphangiogenesis, or a single molecular pathway is likely to lead to failure in terms of therapeutic development.

Many questions remain unanswered: What are the stages of pre-metastatic development in LNs? Which significant element could be utilized for tumor diagnosis and/or to assess the effect that tumors have on the development of LN and distant metastases? It is also essential to identify the optimal therapeutic molecular target(s) and the factors that are critical for cross-talk between tumors and LNs.

Although accumulating studies have prompted the understanding of LN pre-metastatic niche formation in HNSCC, many problems need to be further elucidated. Further research is needed to elucidate the basic mechanisms/characteristics of anti-lymphangiogenesis in HNSCC. Due to the key role of lymphangiogenesis in pre-metastatic niche formation, more researches are needed in this field to explore the potential of anti-lymphangiogenesis in HNSCC treatment, which can support new strategy for the patients.

## Author Contributions

CH: Conceptualization, Writing - original draft, Writing - review and editing. QH: Conceptualization, Writing - original draft, Writing - review and editing. QS: Conceptualization, Resources, Supervision, Writing - review and editing. All authors contributed to the article and approved the submitted version.

## Conflict of Interest

The authors declare that the research was conducted in the absence of any commercial or financial relationships that could be construed as a potential conflict of interest.

## Publisher’s Note

All claims expressed in this article are solely those of the authors and do not necessarily represent those of their affiliated organizations, or those of the publisher, the editors and the reviewers. Any product that may be evaluated in this article, or claim that may be made by its manufacturer, is not guaranteed or endorsed by the publisher.

## Abbreviations

LN, lymph node; LNM, Lymph node metastasis; EVs, extracellular vesicles; HNSCC, head and neck squamous cell carcinoma; LV, lymphatic vessel; SLN, sentinel lymph node; VEGFs, vascular endothelial growth factors; LECs, lymphatic endothelium cells; FN1, Fibronectin 1; SIRT2, sirtuin 2; HNC, head and neck cancer; Ang-1, angiopoietin-1; OSCC, oral squamous cell carcinoma; IGF, insulin-like growth factor; sEVs, small extracellular vesicles.
